# Accuracy of Palatal Orthodontic Mini-Implants Placed Using Fully Digital Planned Insertion Guides: A Cadaver Study

**DOI:** 10.3390/jcm12216782

**Published:** 2023-10-26

**Authors:** Lea Stursa, Brigitte Wendl, Norbert Jakse, Margit Pichelmayer, Frank Weiland, Veronica Antipova, Barbara Kirnbauer

**Affiliations:** 1 Department of Dental Medicine and Oral Health, Division of Oral Surgery and Orthodontics, Medical University of Graz, Billrothgasse 4, 8010 Graz, Austria; brigitte.wendl@medunigraz.at (B.W.); norbert.jakse@medunigraz.at (N.J.); margit.pichelmayer@medunigraz.at (M.P.); barbara.kirnbauer@medunigraz.at (B.K.); 2 Private Practice, Untere Schmiedgasse 16, 8530 Deutschlandsberg, Austria; frank.weiland@medunigraz.at; 3 Division of Macroscopic and Clinical Anatomy, Gottfried Schatz Research Center, Medical University of Graz, Auenbruggerplatz 25, 8036 Graz, Austria; veronica.antipova@medunigraz.at

**Keywords:** orthodontics, mini-implant, temporary anchorage device, guided surgery, surgical template, CBCT, intraoral scan, digital workflow, CAD/CAM, transfer accuracy

## Abstract

Digital workflows have become integral in orthodontic diagnosis and therapy, reducing risk factors and chair time with one-visit protocols. This study assessed the transfer accuracy of fully digital planned insertion guides for orthodontic mini-implants (OMIs) compared with freehanded insertion. Cone-beam computed tomography (CBCT) datasets and intraoral surface scans of 32 cadaver maxillae were used to place 64 miniscrews in the anterior palate. Three groups were formed, two using printed insertion guides (A and B) and one with freehand insertion (C). Group A used commercially available customized surgical templates and Group B in-house planned and fabricated insertion guides. Postoperative CBCT datasets were superimposed with the planning model, and accuracy measurements were performed using orthodontic software. Statistical differences were found for transverse angular deviations (4.81° in A vs. 12.66° in B and 5.02° in C, *p* = 0.003) and sagittal angular deviations (2.26° in A vs. 2.20° in B and 5.34° in C, *p* = 0.007). However, accurate insertion depth was not achieved in either guide group; Group A insertion was too shallow (−0.17 mm), whereas Group B insertion was deeper (+0.65 mm) than planned. Outsourcing the planning and fabrication of computer-aided design and computer-aided manufacturing insertion guides may be beneficial for certain indications; particularly, in this study, commercial templates demonstrated superior accuracy than our in-house–fabricated insertion guides.

## 1. Introduction

Since their introduction by Gainsforth and Higley in 1945 [1], orthodontic mini-implants (OMIs) have expanded the treatment options in orthodontics, serving as temporary anchorage devices (TADs) for intraoral anchorage reinforcement and proving superior to conventional methods [2]. Their advantages include reduced need for patient compliance, improved anchorage, cost-effectiveness, and ease of insertion and removal, making them essential in modern orthodontics [3].

Despite these advantages, risks such as nerve involvement, bleeding from blood vessel trauma, and perforation into the nasal cavity or maxillary sinus persist [4]. Therefore, precise preoperative planning is crucial to minimize these risks and ensure accurate OMI placement [5].

The field of dentistry has shown increasing interest in three-dimensional (3D) technologies in recent years, revolutionizing the orthodontic treatment planning process [6]. Cone-beam computed tomography (CBCT) has enabled better preoperative planning by providing a 3D view of adjacent anatomical structures [7]. Dedicated software allows virtual implant position planning and the creation of corresponding surgical guides. With advancements in 3D technologies, insertion templates and digitally designed orthodontic appliances can be “computer-aided design and computer-aided manufacturing” (CAD/CAM)-fabricated without analog in-between steps [8]. Surgical templates aim to achieve a one-visit protocol, enhancing clinician precision and reducing chair time [9]. CBCT-based insertion guides are particularly beneficial for patients with challenging anatomical situations, such as cleft palate, impacted teeth, or minor palatal bone support [10]. Based on the virtual position of planned OMIs, an individual orthodontic appliance can be designed and CAD/CAM-printed, enabling the installation of miniscrews and orthodontic devices in a single appointment, thereby reducing patient chair time and costs [11]. In this context, some recent studies investigated the transfer accuracy of different insertion guides, although not all of them applied a solely digital workflow, as presented in this study [5,12,13,14]. Möhlhenrich et al.’s cadaveric study [12] showed that tooth-supported silicone guides were superior in terms of transfer precision compared to gingiva-supported guides, with sagittal angular deviations of 3.67° (SD 2.25) vs. 6.46° (SD 5.5). The study by Ludwig et al. [13] investigating “sterile” and “nonsterile” CAD/CAM insertion guides demonstrated that heat treatment during the sterilization process improved transfer accuracy and achieved a clinically acceptable mean deviation of 0.81% at the coronal distance of the mid-mini-implant head. Pozzan et al. [14] analyzed the influence of different steps of the digital workflow on the deviation of the OMI’s axis of guided insertion and showed that the laboratory step resulted in significantly lower axial deviations (2.12° ± 1.62°) than the clinical step (6.23° ± 3.75°).

This anatomical study compares two different insertion guides, both fabricated within a purely digital workflow, and investigates their precision to determine if one method may be preferable. The data gathered during this study allow comparison with conventional freehand OMI insertion.

## 2. Materials and Methods

Ethical approval for this study was obtained from the ethics committee of the Medical University of Graz (EK 32-550 ex 19/20).

While alive, all body donors provided informed consent for the donation of their postmortem tissues for research. All body donors were bequeathed to the Division of Macroscopic and Clinical Anatomy of the Medical University of Graz under the approval of the Anatomical Donation Program of the Medical University of Graz and in accordance with Austrian laws concerning body donation.

This study examined 32 human cadaver heads, provided by the Division of Macroscopic and Clinical Anatomy of the Medical University of Graz, with intact palatal gingiva that were embalmed using a modified Thiel technique [15]. These body donor skulls were randomly divided into three groups for OMI insertion. In Group A (n = 12), a commercially available and individually prefabricated surgical guide (Accuguide^®^; Forestadent Bernhard Förster GmbH, Pforzheim, Germany) was used. In Group B (n = 12), self-designed, individually adapted 3D-printed insertion templates were used. In contrast, in Group C (n = 8), a surgical guide was not used, with the TADs being placed conventionally in a freehanded manner. In total, 64 orthodontic mini-implants (1.7 × 8.0 mm; OrthoEasy^®^ Pal; Forestadent Bernhard Förster GmbH), with 2 OMIs (right and left OMIs) per body donor head, were inserted in the anterior palate, lateral to the palatal suture by an experienced oral surgeon.

### 2.1. Digital Planning and Clinical Procedure

The digital planning and clinical procedure involved obtaining a CBCT dataset (Planmeca Promax 3D Max^®^, Helsinki, Finland) with dimensions of 10.0 × 9.3 cm (diameter × height) and a voxel size of 200 µm, along with an intraoral scan of the upper jaw (Trios 3^®^; 3Shape, Copenhagen, Denmark) for each body donor head.

In Group A, digital imaging and communications in medicine (DICOM) data and a surface standard triangulation language (STL) file for each skull were uploaded to the encoded professional planning portal (Forestadent Bernhard Förster GmbH). OMI positioning was performed by an internationally renowned TAD expert and digital dentistry pioneer. After final approval of the positioning proposal, the printed insertion guide and a resin-printed 3D model were delivered.

**Figure 1:**
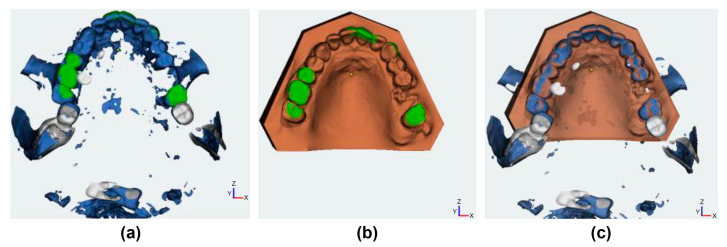
Preoperative superimposition using Onyxceph: (**a**) CBCT dataset; (**b**) intraoral surface scan model; (**c**) superimposition (based on green surfaces **a** + **b**) of both datasets.

For Group B, the DICOM data were uploaded into specific orthodontic planning software (Onyxceph; Image Instruments GmbH, Chemnitz, Germany) and matched with the corresponding STL file of the intraoral scan of the upper jaw (Figure 1).

After superimposition, screw positions were virtually planned using the TADmatch module of Onyxceph, selecting the same TADs from a virtual library used in the clinical procedure. The virtual OMIs were positioned based on the planned orthodontic device and adjacent anatomical structures. Two mini-implants (1.7 × 8 mm; OrthoEasy^®^ Pal; Forestadent Bernhard Förster GmbH, Pforzheim, Germany) with a 6 or 7 mm interscrew distance were planned in a paramedian position at an angle of approximately 90° to the palatal plane. The STL file of the positioning model was exported to Appliance Designer^®^ software (3Shape), where the dental technician virtually designed the surgical guides. The guides were then 3D-printed using a light processing technique (Asiga Pro2^®^; Dentona, Dortmund, Germany) and subjected to light hardening (Otoflash G171^®^; Dentona) with 2 × 2000 flashes in the presence of nitrogen (N_2_) gas. The aim was to create a reduced-size template with a more skeletonized design compared with previous insertion guides.

During OMI insertion, the surgical guides were positioned on the teeth and held in place by an assistant, and the oral surgeon performed the insertion without predrilling.

In Group C, the oral surgeon examined the initial records and performed conventional freehanded manual insertion, as typically conducted during the clinical routine.

**Figure 2:**
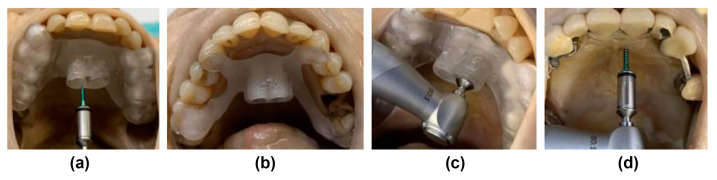
Insertion process of all groups: (**a**) Accuguide^®^ and drill guide with OMI; (**b**) in-house–fabricated insertion guide; (**c**) integrated automatic stop; (**d**) conventional freehanded insertion.

A contra-angle handpiece drive (Prosthodontic Implant Driver; W&H, Bürmoos, Ignaz-Glaser Str. 53, 5111 Bürmoos, Austria) with a corresponding screwdriver was used for screw insertion without predrilling in all groups. Torque was limited to 35 Ncm. Using the surgical templates, the insertion automatically stopped when the screwdriver contacted the top edge of the insertion guide (Figure 2).

### 2.2. Software Analysis and Accuracy Measurements

**Figure 3:**
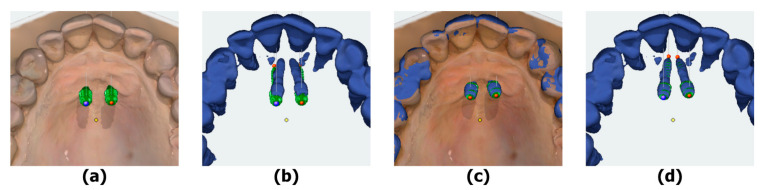
Postoperative superimposition and measurements (e.g., blue mark = “Point 1”): (**a**) presurgical planning model with digital OMIs; (**b**) digital OMIs and postoperative CBCT dataset; (**c**) postsurgical superimposition; (**d**) digital OMIs matched with the postsurgical CBCT dataset.

Postoperatively, CBCT scans (Planmeca Promax 3D Max^®^, Helsinki, Finland) were performed on each body donor head using the same settings applied preoperatively. The postoperative CBCT data were then matched with the corresponding preoperative planning data using the Register3D tool in Onyxceph. Dental reference points on each model were used for superimposition of the datasets. Once the pre- and postsurgical models were aligned, the actual OMI positions from the obtained postsurgical CBCTs were superimposed with the virtual OMIs using an iterative closest point algorithm for automatic surface registration (Figure 3).

Linear measurements were conducted to evaluate the insertion depth and to measure deviations and interscrew distances at the head and tip of the screw. Angular measurements were performed to assess the parallelism of the OMIs and their deviations compared with the preoperatively planned positions. All superimposition and measurements were performed three times by a single experienced orthodontist.

In Group C, postoperative measurements were directly performed in the CBCT software (Planmeca Romexis^®^, Helsinki, Finland). The axes of the OMIs were defined, and sagittal and transversal angles were measured to examine OMI parallelism. These angular deviations between left and right miniscrews were compared with guided insertion.

### 2.3. Statistical Analysis

Statistical analysis was performed using the software package SPSS version 27.0 (IBM SPSS Statistics, IBM Corporation, Armonk, New York, NY, USA). Continuous variables were presented as means ± standard deviations. The independent Student’s *t*-test was used to compare OMI position data, and analysis of variance was performed for comparison among the three groups. The significance level was set at *p* ≤ 0.05.

With a sample size of 12 in each of the two main groups, the study had a power of 87.71% to detect a difference in means of −0.4 (the difference between a Group 1 mean, µ_1_, of 0.56 and a Group 2 mean, µ_2_, of 0.96), assuming a common standard deviation of 0.3, using a two-group *t*-test with a 5% two-sided significance level.

## 3. Results

Preoperatively, all cases exhibited parallelism between right and left OMIs, but postoperatively, none of the investigated miniscrew pairs were exactly parallel. The deviation from parallelism was measured as angles between both implant axes, and it was significantly smaller (*p* = 0.030) in Group A (5.19° ± 2.71°) than in Group B (10.41° ± 7.29°).

There were no significant differences between the left and right OMIs in both groups (right OMI: *p* = 0.720; left OMI: *p* = 0.206). However, in Group A, the angular deviation was higher for the right miniscrew (4.68° ± 1.77°), whereas in Group B, the left OMI exhibited higher deviations (6.66° ± 6.13°). When considering both groups together, no significant differences were found between the left and right mini-implants (*p* = 0.698).

**Figure 4:**
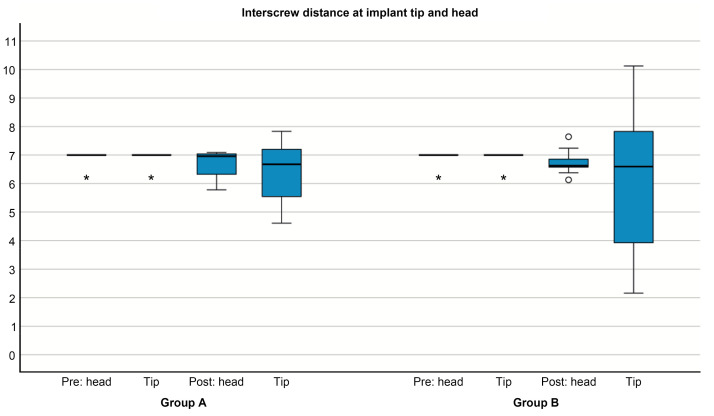
Interscrew distance (mm) measured at the implant tip and head levels pre- and postoperatively in Groups A and B. Preoperatively, in both groups, at least 50% had an interscrew distance of 7 mm, and all other measurements were detected as extreme outliers (*). The postoperative dispersion was larger and less symmetrical for tip measurements compared to measurements at the OMI head. Two mild outliers (°) were detected in the measurement group “post head”.

Mean interscrew distances, measured at the implant tip and head, became smaller between the planned and actual positions in both groups, but these changes were not statistically significant (Figure 4; Table 1). The actual implant head positions in both groups were found to be closer to the median palatal suture (Group A: 0.30 ± 0.22 mm; Group B: 0.27 ± 0.18 mm) compared with the virtually planned implants.

As presented in Table 2, the mean deviations between virtual and actual implant positions were higher in Group B for both OMIs, both at the level of the implant tip and head. These differences were statistically significant for the right OMI at the level of the implant head (0.90 ± 0.37 mm) and for the left OMI, both at the head level (0.96 ± 0.46 mm) and the implant tip (1.43 ± 0.82 mm).

Significant statistical differences were also observed for vertical deviations measured at the implant head (right OMI: *p* = 0.002; left OMI: *p* < 0.001). In Group A, the planned insertion depth of the implants (−0.17 mm) could not be achieved, whereas in Group B, the performed insertion was deeper (+0.65 mm) than planned.

In terms of transverse angular deviations between right and left OMIs for Groups A and B, Group A demonstrated significantly higher implant axis accuracy (4.81° ± 4.09°; *p* = 0.028). However, no statistical difference was found for sagittal angular deviations (*p* = 0.929), with mean angular deviations of 6.42° ± 2.26° in Group A and 5.10° ± 2.20° in Group B.

**Figure 5:**
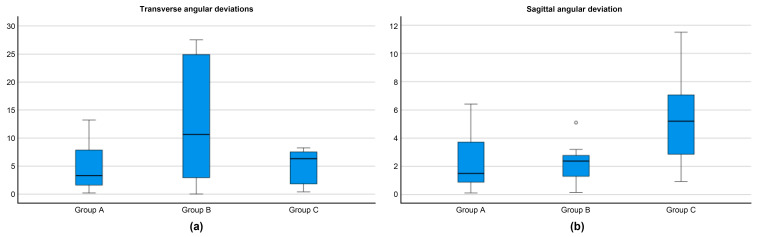
Comparison of transfer accuracy among Groups A, B, and C: (**a**) transverse angular deviations; (**b**) sagittal angular deviations. One mild outlier (°) was detected for sagittal angular deviations in Group B.

Figure 5 illustrates the comparisons among Groups A, B, and C for sagittal and transverse angular deviations between right and left mini-implants. Transverse angular deviations were significantly less accurate in Group B than in the other groups (*p* = 0.003), whereas sagittal angular deviations were significantly higher in Group C than in the other groups (*p* = 0.007).

## 4. Discussion

The primary focus of this study was to compare guided miniscrew insertion using the applied digital workflow. The goal was to assess the accuracy and reliability of the virtual planning process with two different types of insertion guides and compare it to a control group with conventional freehand OMI insertion. Although Group A demonstrated better results, significant differences were found for all methods (Table 1; Figure 5). Deviations from the digitally planned positions were mostly within tenths of millimeters, and axial deviations were generally smaller than six degrees.

In contemporary dentistry, 3D imaging has become integral to various dental disciplines [16]. Intraoral surface scans have replaced conventional impressions owing to their increased precision, and CBCTs provide 3D radiologic information with lower radiation doses compared with computed tomographies [17]. The use of intraoral surface scans allows for the creation of precise surface models of teeth and gums, which streamline workflows. Research over recent years has shown that digital workflows in dentistry are at least as precise and effective as traditional methods [18].

CBCTs have become an essential diagnostic tool, especially in surgical dentistry, as they accurately display the 3D shape and position of teeth and jaw bones [19]. Cephalometric X-rays, routinely used in orthodontic practice, have limitations as they are two-dimensional (2D) projections of 3D anatomical structures, resulting in image distortion and overlapping of bilateral structures [20]. Owing to the low radiation dose and excellent spatial resolution of CBCTs, dentists can improve diagnosis and use the additional information for treatment planning.

The anterior palate has become a favored insertion site for OMIs owing to the abundance of cortical bone and available attached gingiva [21]. Studies, such as that of Jung et al. [22], have shown that lateral cephalometry provides a reliable assessment of the quantity of vertical bone for paramedian TAD insertion. In the present study, 3D imaging was used for accuracy measurements and to evaluate interferences with surrounding anatomical structures. However, in the clinical routine, an approach such as that of Maino et al. [23], which merges lateral cephalograms and digital dental models to plan and produce surgical guides, might be sufficient. Using lateral cephalograms comes with lower costs and reduced radiation exposure [23], and these 2D X-rays remain part of the basic radiological investigation and represent the gold standard of imaging in patients undergoing orthodontic therapy [22].

Previous studies have investigated various insertion guides, concluding that their use improves TAD insertion accuracy and stability while reducing the failure rate of mini-implants [5,10,12,13,24,25,26,27,28,29]. Tooth-supported guides, rigidly printed and supported on the edges of teeth, were found to ensure higher insertion precision compared with solely gingiva-supported templates [5,12]. Based on these findings, we chose to investigate only tooth-supported guides. Our measurements were performed following the approach of Casetta et al. [24], which involves comparing pre- and postsurgical CBCTs to calculate angular, coronal, and apical deviations between virtually planned and actual OMI positions.

CAD/CAM appliance design relies on virtually planned TAD positions provided by insertion guides, leading to a simplified and improved collaboration with the laboratory, a faster workflow, and more predictable appliances. The simultaneous digital manufacturing of the insertion guide and the superstructure enables a one-visit protocol for the insertion of mini-implants and orthodontic appliances [30,31]. However, achieving accurate TAD insertion is crucial for a successful one-visit protocol [32], particularly when an increased number of miniscrews and a more rigid orthodontic appliance are required. Exceeding a certain tolerance level for angular deviations may necessitate a two-step protocol [33]. Minor errors in the digital workflow or during clinical implementation can accumulate and result in deviations of the mini-implants, potentially leading to misfit of the prepared appliance. Factors such as the coronal distance of the OMIs, insertion depth, apical distance, and the angle between the two TADs are relevant in this context [13]. The direction of the deviation may also play a role, as TAD deviations can potentially compensate for each other in some favorable cases [14]. A systematic review investigating the accuracy of surgical guides for dental implantology found mean angular deviations of 3.5° and mean vertical deviations of 1.2 and 1.4 mm at the implant head and implant tip, respectively, which were deemed clinically acceptable [34].

Although freehand insertion is generally safe and accurate [35], when planning a TAD-supported orthodontic appliance, a second visit becomes necessary because the exact positions of the OMIs cannot be predefined.

Comparing workflows with and without surgical templates, similar interscrew parallelism can be achieved. It has been shown that OMIs may not remain absolutely stationary and can experience angular displacements up to 11° per OMI due to orthodontic loading [36]. Thus, even if TADs were initially parallel, they may not remain parallel throughout the entire treatment.

The deviation in insertion depth found in our study aligns with the findings of Kniha et al. [5], who attributed this effect to the resilient properties of silicone. The vertical inaccuracies observed in our study may be related to the difference in rigidity between the surgical guides in Group B and those in Group A. The in-house-produced guides in Group B might have been less rigid, resulting in deeper insertion, whereas the other guides were more rigid, making it impossible to reach the final insertion depth. Different printing settings and offsets between the two groups could also contribute to these variations. Tissue rigidity is unlikely to have been relevant in this study, as all guides were designed to be tooth-supported. The fit of the insertion guides in Group A appeared superior, possibly due to their more pronounced extension to the buccal tooth surface compared with Group B, whereas the in-house-produced guides sometimes appeared to lack reliable stability and did not stay in place without the assistance of fixation.

Comparing our study to others is challenging owing to the heterogeneity of reference points and measurement methods among published studies. Differences in anatomical areas, study protocols, and measurement techniques make direct comparisons difficult [9,10,12,14,37].

Despite promising results, this study has limitations. First, the use of embalmed cadaver heads with preserved tissue may not fully represent the real clinical situation, as blood perfusion is lacking, tissue quality and properties change after preservation [5], and bone remodeling is absent [38]. Second, the age difference between the body donors, elderly individuals with multiple missing or filled teeth and even dental implants or prostheses, and the typical orthodontic clientele, mostly adolescents or young adults with complete dentition, is also a limitation [39]. Third, artefacts caused by prosthetic restorations in the cadavers’ CBCT datasets could have influenced superimposition accuracy. The sample size is another limitation of this study, as n = 12 would have been ideal for all three investigation groups. Unfortunately, insufficient appropriate body donors were available at the time of this study. Hence, we opted for n = 8 for Group C, viewing it as an additional group, with groups A and B as the main investigation groups. Finally, hard and soft tissue behavior in the body donors might differ from that in a real clinical situation.

To date, few studies have investigated the transfer accuracy of guided OMI insertion using postoperative CBCTs. To the best of our knowledge, only two such studies have been conducted in a clinical setting. Casetta et al. [24] performed a clinical study comparing presurgical and postsurgical CBCTs of five patients receiving palatal OMIs, whereas Liu et al. [40] investigated surgical guides for interradicular OMI insertion using CT scans. Kniha et al. [5] used presurgical lateral cephalograms and postoperative CBCTs in a body donor study. Bae et al. [10] conducted another cadaver study to examine the transfer accuracy of insertion guides for interradicular OMI placement using CBCTs. To avoid possible sequelae of radiation exposure to living participants, the current study followed this precedent and limited the analysis to embalmed specimens. The next step would be a clinical study examining the precision of surgical guides, ideally incorporating alternative imaging methodologies with reduced radiation exposure. CBCT-based investigations of transfer accuracy are clinically relevant as scanbody measurements focus on the oral portion, which may lead to incomplete data regarding the orientation of the implant tip. Precise visualization of the implant tip is crucial for avoiding damage to adjacent anatomical structures in clinical settings.

Although many studies have investigated the accuracy of guided OMI insertion and deemed deviations clinically acceptable, no maximum tolerance level has been defined for deviations in one-visit protocols. Further studies are needed to investigate the required accuracy for simultaneous installation of OMIs and TAD-based appliances and to establish clear tolerance limits for individually manufactured appliance insertion.

## 5. Conclusions

CBCT has become a vital imaging tool in dentistry. Although guided OMI insertion shows slight deviations, it remains an accurate and safe method that is widely used in daily clinical practice. The ability to apply a one-visit protocol is a major advantage of digitally planned insertion guides over freehanded TAD insertion. However, achieving 100% accuracy in delivering the planned TAD position is not always possible. Our study found that treatment results were more reliable using commercially available insertion guides compared with in-house self-fabricated insertion guides, making the former the recommended choice when insertion guides are indicated.

## Figures and Tables

**Table 1:** Interscrew distances (mm) measured at the implant head and implant tip pre- and postsurgically (*: *t*-test for independent samples).

Interscrew Distance		Min	Max	Mean	SD (+/−)	Sign. *p* *
Head pre	Group A	6.00	7.00	6.83	0.39	*p* = 0.557
	Group B	6.00	7.00	6.92	0.29	
Tip pre	Group A	6.00	7.00	6.83	0.39	*p* = 0.557
	Group B	6.00	7.00	6.92	0.29	
Head post	Group A	5.78	7.09	6.71	0.46	*p* = 0.895
	Group B	6.13	7.64	6.74	0.39	
Tip post	Group A	4.61	7.83	6.41	1.10	*p* = 0.719
	Group B	2.16	10.12	6.12	2.57	

**Table 2:** Deviations (mm) between virtual and real implant positions measured at the implant head and implant tip (*: *t*-test for independent samples; significant differences are shown in bold text).

Deviation Pre–Post		Min	Max	Mean	SD (+/−)	Sign. *p* *
Right OMI head	Group A	0.05	1.11	0.60	0.31	*p* = 0.003
	Group B	0.25	1.59	0.90	0.37	
Right OMI tip	Group A	0.18	2.05	0.87	0.43	*p* = 0.091
	Group B	0.20	3.34	1.17	0.76	
Left OMI head	Group A	0.24	1.14	0.56	0.28	*p* = 0.001
	Group B	0.18	1.83	0.96	0.46	
Left OMI tip	Group A	0.27	2.22	0.87	0.55	*p* = 0.008
	Group B	0.56	3.96	1.43	0.82	

## Data Availability

The data that support the findings of this study are available from the corresponding author upon reasonable request. The data are not publicly available owing to ethical restrictions.
